# Outcomes of the addition of oral administration of curcumin-phospholipid to hyaluronic acid-based tear substitute for the treatment of dry eye disease

**DOI:** 10.3389/fopht.2023.1236525

**Published:** 2023-10-05

**Authors:** Massimiliano Borselli, Fausto F. Ferrari, Pietro Bianchi, Costanza Rossi, Giovanna Carnovale Scalzo, Domenica Mangialavori, Vincenzo Scorcia, Giuseppe Giannaccare

**Affiliations:** Department of Ophthalmology, University Magna Graecia of Catanzaro, Catanzaro, Italy

**Keywords:** curcumin, dry eye disease, ocular surface, tear substitutes, tear film

## Abstract

The aim of this study is to report the clinical outcomes of oral supplementation with curcumin-phospholipid in addition to hyaluronic acid-based tear substitute for the management of dry eye disease (DED). Patients with a diagnosis of DED confirmed by pathological values of both NIKBUT <10 s. and OSDI Questionnaire score > 12 were included. Patients were randomized to receive 2 different treatments: 0.25% hyaluronic acid-based tear substitute 3 time daily (Group 1) or as above plus curcumin-phosphatidylcholine complex tablets once a day (Group 2). Patients were evaluated at baseline (T0) and after 90 days of treatment (T1) by means of Keratograph for the measurement of NIKBUT, TMH, meibomian gland dropout and bulbar redness. Overall, data from 90 eyes of 45 patients were included. Group 1 consisted of 48 eyes of 24 patients, while group 2 included 42 eyes of 21 patients. When comparing median values of both groups at T0, no statistically significant differences were found for all parameters; instead for T1, statistically significant differences were found for redness and OSDI compared to Group 1. In group 1, a statistically significant reduction after the treatment was detected for Nikbut average and OSDI questionnaire; while in group 2, a statistically significant reduction after treatment was recorded for Nikbut average, bulbar redness and OSDI questionnaire. The addition of an oral supplement containing curcumin-phospholipid may help in a greater improvement of bulbar redness and subjective ocular symptoms compared to the treatment with tear substitutes alone for the management of DED.

## Introduction

Dry eye disease (DED) is known to be an ophthalmologic disorder frequently seen in routine clinical practice. The Tear Film & Ocular Surface Society (TFOS) Dry Eye Workshop II (DEWS II) recently revised the definition of DED, presenting it as an ocular surface disorder characterized by the combination of tear film instability, hyperosmolarity, and inflammation that disrupts the homeostasis of the ocular system (1, 2). Typical indications and manifestations of DED encompass having a foreign body sensation, impaired visual clarity, excessive tearing, sensitivity to light, and ocular redness. A milestone of the treatment is tear substitutes controlling the symptoms and protecting the ocular surface. Over the years, there has been increasing attention toward the potential benefits of oral dietary supplements for the treatment of DED. These supplements are generally regarded as safe, particularly when used over an extended duration. Over the years, the effects of various nutraceuticals, such as antioxidants (3), anthocyanins (4), carotenoids (5), and omega-3 fatty acids, have been deepened (6–9).

## Characteristics of curcumin

**Figure 1:**
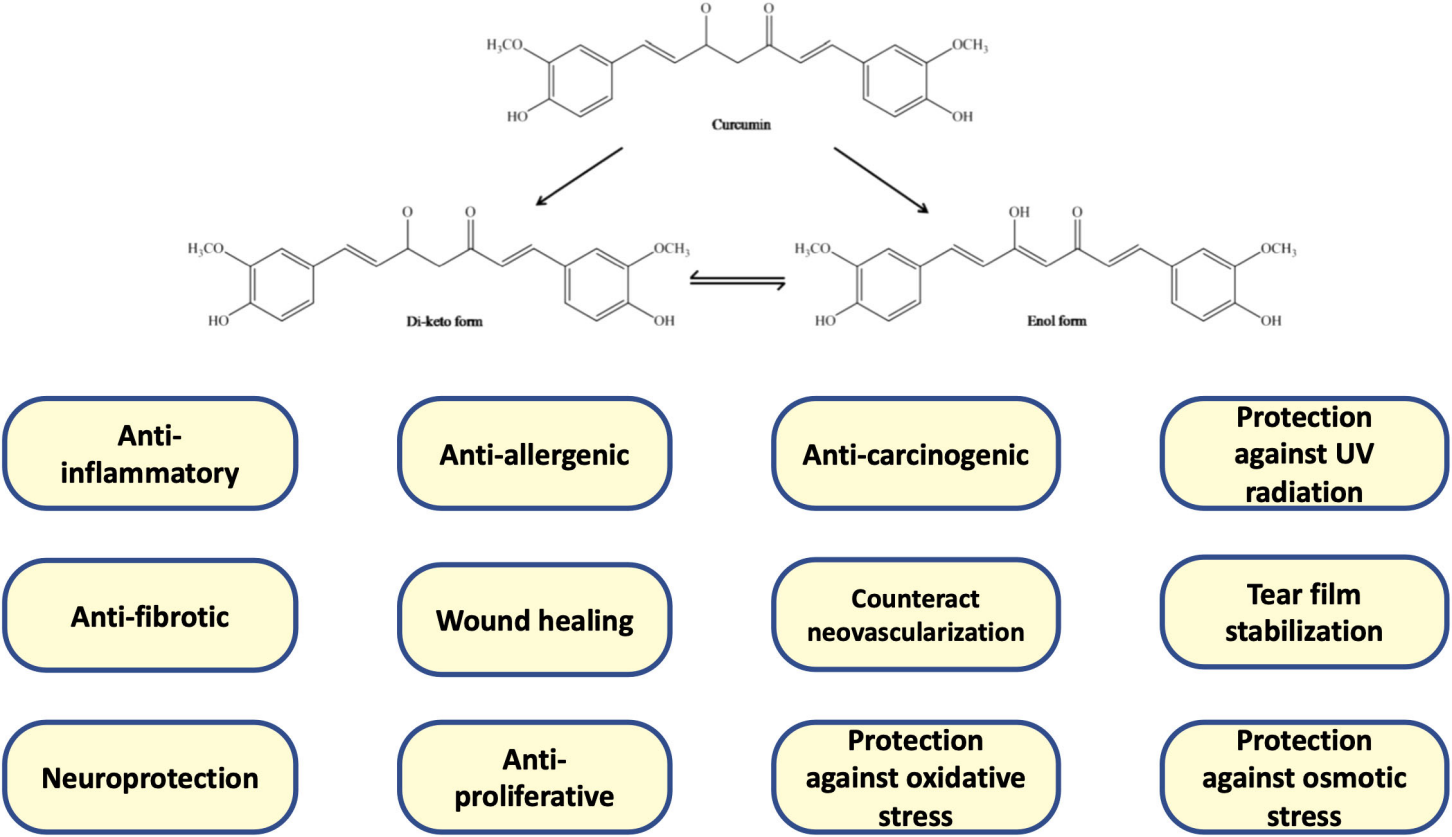
Depiction of the chemical structure of curcumin in its keto and enol tautomeric forms (11). This compound showcases significant beneficial effects on the ocular surface.

Turmeric, derived from the plant *Curcuma longa* and commonly used as a spice in curry dishes, has gained recognition for its medicinal properties. The market value of curcumin, one of the active compounds in turmeric, was estimated to be approximately US$500 million in 2016 (10). The chemical formula for curcumin is C_21_H_20_O_6_. Curcumin can exist in various tautomeric forms, which are structures where the atoms are arranged differently yet they can interconvert while retaining the same chemical formula. The two main tautomers of curcumin are the keto form and the enol form (11). Curcumin has garnered significant attention in recent years for its potential anti-inflammatory, anti-diabetic, anti-cancer, and anti-aging effects (**Figure 1**). Nonetheless, the effectiveness of curcumin as a therapeutic agent is hindered by its low solubility in water, inadequate bioavailability, and unfavorable pharmacokinetic properties. In order to address these limitations, researchers have explored different formulations and delivery systems for curcumin, as documented in scientific literature. These include liposomes, nanoparticles formulated with lipids or polymers, micelles, microemulsions, and even nanoparticles incorporating metals (12). NORFLO^®^ is a food supplement, with a formula based on curcuminoids complexed in phytosomes (iPhytoone, Eye Pharma S.P.A., Genoa, Italy). iPhytoone^®^ contains a minimum of 2% phosphatidylserine, which allows a rapid breakdown of curcumin to enable them to be absorbed from the first minutes after administration. Phytosomes are a transport system for poorly bioavailable active ingredients and are used to improve the bioavailability of active ingredients. They are complexed with different substances, allowing them to overcome the lipid barrier of the alimentary canal and be absorbed.

## Effects of curcumin on ocular surface system

For several years, the interest in curcumin in the field of ophthalmology has grown, and many clinical studies have tested its safety and efficacy in different eye diseases. The inhibition of the basic fibroblast growth factor (bFGF) and vascular endothelial growth factor (VEGF) oppose the pro-angiogenic effect induced by hypoxia and inflammation (13). Furthermore, the utilization of curcumin for enhancing wound healing has indicated its possible application in conditions where there is impaired restoration of the corneal epithelium, such as neurotrophic keratitis. To support this notion, a study conducted by Guo et al. showcased the effectiveness of intranasally administered curcumin nanomicelles in a mouse model of diabetic keratopathy. The use of curcumin has been found to play a role in restoring the balance of the ocular surface by diminishing the presence of reactive oxygen species, suppressing the expression of inflammatory mediators, and enhancing the levels of neurotrophic factors (14, 15). Another potential application for curcumin is in the treatment of DED and various studies have indicated that curcumin showed promising results (15). The purpose of this study is to evaluate the outcomes of using a curcumin-based dietary supplement in addition to tear substitute therapy in the management of DED, through the assessment of symptoms and objective parameters of the ocular surface.

## Methods

The present prospective study was carried out at the Magna Græcia University of Catanzaro from March 2022 to July 2022. The research conducted in this study received approval from the ethics committee (Comitato Etico Regione Calabria—Sezione Area Centro) and was performed in accordance with relevant guidelines and regulations and in accordance with the Declaration of Helsinki of 1964 and its later amendments. Before any procedure, all participants signed a written informed consent form. Patients with a diagnosis of DED confirmed by pathological values of both non-invasive keratograph break-up time (NIKBUT) <10 s and Ocular Surface Disease Index (OSDI) questionnaire score > 12 were included. Patients using glaucoma therapy, contact lenses, or topical corticosteroids were excluded. During the initial screening visit, relevant data regarding gender, age, body weight, BMI, medical history, concurrent medication use, history of allergies, and drug reactions were collected and recorded. Enrolled patients were randomized to receive a tear substitute or curcumin tablets in addition to a tear substitute in a 1:1 ratio by means of computer-generated random number allocation. Patients were divided into two groups according to study treatment: group 1 was treated with 0.25% hyaluronic acid-based tear substitute three times daily (Perflo, Eye Pharma S.P.A., Genoa, Italy). The treatment delivered to group 2 was curcumin tablets once a day (NorFlo, Eye Pharma S.P.A., Genoa, Italy) in addition to a tear substitute three times daily. NorFlo is a capsule containing 100 mg of curcumin and 200 mg of soy-phospholipid. The follow-up protocol provided for each patient in the two groups was a screening visit (T0) that included a complete ophthalmological examination. A non-invasive ocular surface examination was carried out by means of Oculus Keratograph 5M (K5 M; Oculus GmbH, Wetzlar, Germany). The instrument allowed the evaluation of tear meniscus height (TMH), non-invasive keratograph break-up time (NIKBUT), and infrared meibography of the lower eyelid for the calculation of meibomian gland dropout. In brief, TMH was processed on the basis of an infrared image acquired from the center of the lower eyelid. The cut-off value is 20 mm, below which values were considered pathological. NIKBUT was evaluated using the tear break-up time acquired through the reflection obtained from Placido’s discs; a value < 10 s was considered pathological. Meibography was performed on an infrared image of the extroflexed lower eyelid. Its rating is based on Jenvis Meiboscore, which ranks gland dropout using a scale from 0 to 3. Redness is determined based on an infrared image for the evaluation of conjunctival vessels based on the Jenvis redness score. Statistical analysis was performed with GraphPad Prism 8.2.1 (GraphPad Software, Inc., San Diego, CA). Descriptive statistics were computed for all variables. Data were analyzed for each group with the Kolmogorov–Smirnov test, which confirmed non-parametricity. The Mann–Whitney U test was used to compare the two groups values at T0 and T1 (**Table 1**). Delta was calculated for T0 vs T1 for each group to assess the changes on the parameters considered. The criterion for a significance test by treatment was set at *p* < 0.05.

**Table 1:** Baseline characteristic of both groups obtained with the Mann–Whitney U test.

	Median P	*p*-value
TMH	0.27	0.34
NIKBUT	6.59	0.49
NIKBUT average	15	0.79
Redness	1.3	0.80
Meibography	1	0.34
OSDI questionnaire	26.5	0.09

TMH, teat meniscus height; NIKBUT, non-invasive keratograph

break-up time; OSDI, Ocular Surface Disease Index.

## Results

Overall, 48 patients were enrolled at the beginning of the study. Out of these, three patients belonging to group 2 did not complete the follow-up and were excluded from the analysis. Therefore, data from 90 eyes of 45 patients who completed the entire follow-up were included. Group 1 consisted of 48 eyes of 24 patients (15 males, 9 females; mean age 62.87 ± 14.83 years), while group 2 included 42 eyes of 21 patients (9 males, 12 females; mean age 59.25 ± 13.13 years). The two groups had the same baseline characteristics (*p* = 0.34 for TMH; *p* = 0.489 for NIKBUT; *p* = 0.798 for average NIKBUT; *p* = 0.801 for redness; *p* = 0.335 for meibography; and *p* = 0.099 for OSDI). In group 1, a statistically significant improvement after the treatment was detected for the average NIKBUT (12.03 s ± 5.94 s (SD) at T0 vs 15.45 s ± 4.77 s (SD) at T1; *p* = 0.0004) and OSDI (27.76 s ± 14.33 s (SD) at T0 vs 17.72 s ± 7.21 s (SD) at T1, *p* < 0.0001); in group 2, a statistically significant improvement after treatment was recorded for the average NIKBUT (12.33 s ± 6.04 s (SD) at T0 vs 16.44 s ± 3.92 s (SD) at T1; *p* = 0.0005), bulbar redness (1.301 ± 0.556 (SD) at T0 vs 0.96 ± 0.27 (SD) at T1; *p* = 0.003), and OSDI (35.68 ± 17.23 (SD) at T0 vs 13.86 ± 5.84 (SD) at T1, *p* < 0.0001). When comparing median values of both groups at T1, statistically significantly better values were found in group 2 than group 1 for redness (group 1 median = 1.2 vs group 2 median = 1.0; *p* < 0.001) and OSDI (group 1 median = 16 vs group 2 median = 12.5; *p* = 0.012).

When comparing the difference (delta, Δ) between T0 and T1 for each parameter within the two groups, a statistically significantly higher improvement was found in group 2 than group 1 for TMH (Δ group 1 = 0.026 vs Δ group 2 = –0.7; *p* < 0.0001), redness (Δ group 1 = 1.29 vs Δ group 2 = –2.16; *p* < 0.0001), average NIKBUT (Δ group 1 = 11.42 vs Δ group 2 = –27.25; *p* < 0.0001), and OSDI (Δ group 1 = 12.25 vs Δ group 2 = 21.73; *p* = 0.0014). Conversely, there were no statistically significant differences for NIKBUT (p = 0.3057).

## Discussion

Curcumin is a natural compound widely used as a supportive therapy in various local and systemic pathological conditions, such as respiratory, hepatic, and dermatologic disorders (12). Its potential benefits have been supported by numerous laboratory studies and clinical trials (7, 12). In addition, curcumin has been investigated for its potential use in treating symptoms of ocular discomfort (16). The inflammation of the ocular surface can be mitigated in DED by the use of natural substances with an anti-inflammatory effect, such as curcumin (7, 16). Some studies showed that the use of curcumin supplements alone can reduce the signs and symptoms of DED as well as the frequency of tear substitute usage (6, 16). It has been observed that curcumin exhibits the ability to inhibit the expression of pro-inflammatory cytokines, such as interleukin (IL)-4 and IL-5, in the conjunctiva of mice induced with ovalbumin (17). The effect of curcumin in counteracting the increase in the concentrations of IL-1β, IL-6, and TNF-α induced by hyperosmotic stress has also been studied in human corneal epithelial cells (18). Radkar et al. tested the efficacy of curcumin tablets as a replacement for tear substitutes for DED management and demonstrated significant improvements in the results of Schirmer’s test, OSDI, TBUT, SPEED, ocular staining scores, tear osmolarity, and matrix metallopeptidase 9 (MMP-9), and reduced frequency of artificial tear use against a placebo (16). In the present paper, both therapies enabled an improvement in the management of signs and symptoms of DED. Patients who used only hyaluronic acid-based tear substitutes had a statistically significantly improved NIKBUT and OSDI, while for those who also took oral curcumin there was a statistically significant improvement not only in NIKBUT and OSDI but also in redness. Furthermore, at the end of the 3-month treatment regime, bulbar redness and ocular symptoms were significantly better in the latter group. According to delta values from baseline to last follow-up, the analysis demonstrated higher values for patients who were administered curcumin orally in addition to tear substitute therapy than for those who were administered tear substitutes alone. These data highlight the utility of integrating the standard treatment with tear substitutes with oral natural supplements in the setting of DED. It should be pointed out that one of the study’s limitations was the absence of data regarding medium- to long-term follow-up after the discontinuation of oral therapy.

In conclusion, our study has shown that the addition of a curcumin-based supplement in DED therapy may help in facilitating a greater improvement in ocular discomfort symptoms than tear substitutes alone, especially in severe cases. Eye redness and symptoms perceived by the patients were the parameters mainly affected by the combined treatment of tear substitutes and a curcumin-based supplement. Despite the promising results of the oral formulation of curcumin-phospholipids for DED, further studies are needed to evaluate adherence to treatment in the long-term, particularly in patients who are on polytherapy (19).

## Data availability statement

The raw data supporting the conclusions of this article will be made available by the authors, without undue reservation.

## Ethics statement

The studies involving humans were approved by Comitato Etico Regione Calabria—Sezione Area Centro. The studies were conducted in accordance with the local legislation and institutional requirements. The participants provided their written informed consent to participate in this study.

## Author contributions

All authors listed have made a substantial, direct, and intellectual contribution to the work and approved it for publication.
